# Utility of Thyroglobulin measurement in fine-needle aspiration biopsy specimens of lymph nodes in the diagnosis of recurrent thyroid carcinoma

**DOI:** 10.1186/1742-6413-5-1

**Published:** 2008-01-31

**Authors:** Zubair W Baloch, Julieta E Barroeta, Janet Walsh, Prabodh K Gupta, Virginia A LiVolsi, Jill E Langer, Susan J Mandel

**Affiliations:** 1 Department of Pathology and Laboratory Medicine, University of Pennsylvania Medical Center, 3400 Spruce Street, Philadelphia, PA 19104, USA; 2 Department of Medicine, Division of Endocrinology, University of Pennsylvania Medical Center, 3400 Spruce Street, Philadelphia, PA 19104, USA; 3 Department of Radiology, University of Pennsylvania Medical Center, 3400 Spruce Street, Philadelphia, PA 19104, USA

## Abstract

**Introduction:**

The most common site for the metastasis of papillary carcinoma of the thyroid (PTC) is regional lymph nodes. Ultrasound (US) imaging may identify abnormal appearing lymph nodes, suspicious for PTC recurrence. Although fine needle aspiration biopsy (FNAB) of abnormal lymph nodes is often diagnostic of recurrence, small or cystic lymph nodes may be non-diagnostic due to lack of tumor cells. The measurement of thyroglobulin (TG) levels in FNAB specimens from lymph nodes suspicious for recurrent PTC can serve as an adjunct to the cytologic diagnosis.

**Materials and methods:**

115 abnormal appearing lymph nodes were aspirated under ultrasound guidance in 89 patients with history of thyroid carcinoma. In addition to obtaining material for cytologic interpretation, an additional aspirate was obtained by FNAB and rinsed in 1 ml of normal saline for TG level measurements.

**Results:**

The cytologic diagnoses included: 35 (30%) reactive lymph node, no tumor seen (NTS), 39 (34%) PTC, 23 (20%) inadequate for evaluation due to lack of lymphoid or epithelial cells (NDX) 15 (13%) atypical/suspicious for PTC, and 3 (3%) other (e.g. paraganglioma, poorly differentiated carcinoma and carcinoma not otherwise specified). TG levels were markedly elevated (median 312 ng/ml; normal < 10 ng/ml) in 28 (72%) cases of PTC lymph node recurrence identified on cytology. TG measurements were also elevated in 5 lymph nodes classified as NTS and 4 NDX on cytology which resulted in 5 and 3 carcinoma diagnoses respectively on histological follow-up. Of the 9 atypical/suspicious cases with elevated TG levels all resulted in carcinoma diagnoses on follow-up.

**Conclusion:**

The measurement of TG in FNAB specimens from lymph node in patients with history of PTC is useful in detecting recurrent disease, especially in cases when the specimen is known to be or likely to be inadequate for cytologic evaluation.

## Introduction

Papillary thyroid carcinoma (PTC) is the most common malignant tumor of thyroid. Though in majority of cases PTC behaves in an indolent fashion, it can spread through lymphatics within the thyroid gland (multi-focal disease) and metastasize to the cervical lymph nodes [1-3]. In patients with a previous diagnosis of PTC, the clinical or radiologic detection of enlarged cervical lymph nodes raises the suspicion of recurrent disease [3,4].

Fine needle aspiration biopsy (FNAB) can effectively diagnose PTC because its diagnosis is based upon the demonstration of classic nuclear features [5,6]. It has been shown that FNAB is effective alone or in conjunction with ultrasound guidance in the diagnosis of metastasis from PTC to the cervical lymph nodes [7,8]. However, in some instances the metastatic deposits of PTC in the lymph nodes may undergo degeneration and cystic change [9-11]. In such instances the FNAB of lymph nodes even with ultrasound guidance may only show colloid type material, cellular debris and macrophages without any identifiable tumor cells. These specimens are usually classified as "non-diagnostic or unsatisfactory for evaluation [9,11,12]."

Measurement of thyroglobulin (TG) in the FNAB needle rinse from the cervical lymph nodes has been employed for detecting recurrent thyroid cancer in patients who have undergone total thyroidectomy and ^131^I therapy for differentiated thyroid cancer [11-19].

In this study we evaluated the role of FNAB-TG in detecting lymph node recurrence in patients with previous history of thyroid carcinoma at our institution.

## Materials and Methods

### Patients

The cohort included 115 FNAB specimens in eighty-nine patients (21 male, 68 female; ranging in age 14–83 years (mean: 46.7 years). Seventy-four patients had a history of PTC, two had a history of follicular carcinoma with associated papillary microcarcinoma, one had Hurthle cell carcinoma with associated papillary microcarcinoma and one had history of poorly differentiated carcinoma (PDCA) arising in association with follicular variant of papillary thyroid carcinoma (FVPTC). Surgical pathology slides were not available for review in eleven patients with history of PTC. All patients with previous diagnoses of PTC had been treated by total or partial thyroidectomy with subsequent completion thyroidectomy and ^131^I ablation. Of the 77 PTC (including 3 papillary microcarcinoma), 53 were classified as classic variant, 10 as FVPTC, 12 as tall cell variant of papillary thyroid carcinoma (PTC-TCV), and 2 as diffuse sclerosis variant (PTC-DSV). The tumor size of the cases of PTC was available in 53 cases and ranged from 0.4–7.8 cm (mean 2.07 cm); vascular invasion was seen in 29; extra-thyroidal extension in 36 and multi-focal disease in 41 patients. Lymph node metastases were present at the time of initial surgery in 64 patients.

### FNAB

**Figure 1:**
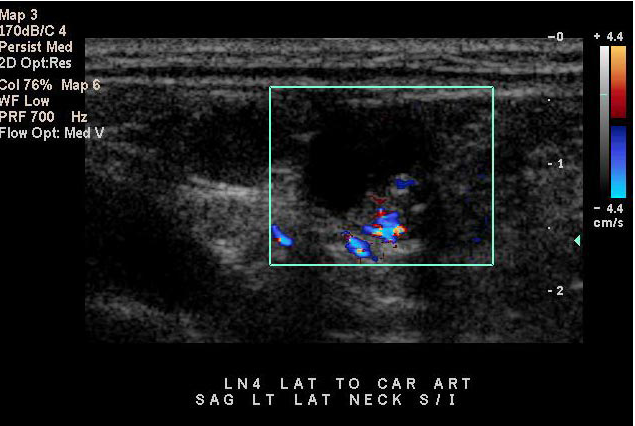
Ultrasound image demonstrates a mixed cystic and solid appearing lateral cervical lymph node in a patient with previous thyroidectomy for papillary thyroid carcinoma.

All lymph nodes were initially localized and measured (size range 0.7–3.7 cm) using a high-frequency linear 10-5 MHz transducer and an ATL 3000 ultrasound scanner (Advanced Technology Laboratories, Bothell, WA) (Fig 1). Ultrasound-guided FNAB was performed using a 25-gauge, 1.5 inch needle and an 8-5 MHz curvilinear transducer using real-time, free hand technique.

The first FNAB pass was evaluated on-site by the cytopathologist; it was divided between fresh smears and Normosol^® ^for Millipore-filter^® ^preparations. Half of the fresh smears were stained with Diff-Quik for immediate on-site evaluation and the others were placed in 95% alcohol for Papanicolaou staining. The second FNAB pass was washed in Normosol and the entire wash was submitted for TG assay.

### TG Assay

TG levels in the lymph node FNAB specimens were measured by employing Nicohls Chemiluminescence Thyroglobulin-TG assay (Nicohls Institute Diagnostics, San Clemente, CA) with an analytical sensitivity of ≤ 0.04 ng/ml and functional sensitivity of ≤ 0.3 ng/ml.

## Results

### FNAB Findings

**Figure 2:**
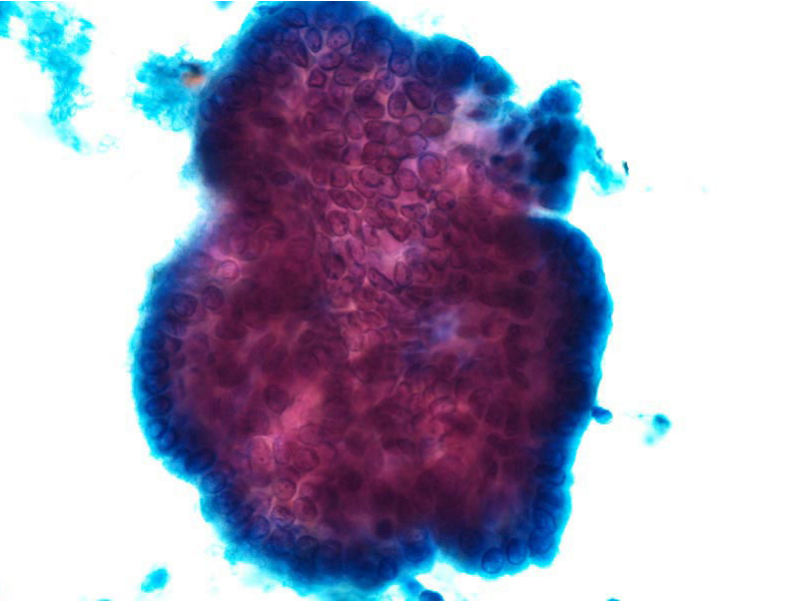
Papanicolaou-stained fine-needle aspiration smear from a lymph node showing metastatic papillary thyroid carcinoma.

Metastatic PTC was diagnosed in 39 (34%) specimens (Fig 2). Fifteen aspirates (13%) were diagnosed as suspicious for PTC; these included 9 cases showing colloid and macrophages and no viable tumor cells present and 6 cases with atypical cell present suspicious for PTC. Thirty-five (30%) cases were diagnosed as reactive lymphoid tissue only no tumor seen, 23(20%) were classified as inadequate for evaluation due to lack of any lymphoid or epithelial cells, and in 3 (3%) cases the diagnosis included paraganglioma 1, PDCA 1 and carcinoma not further specified 1 case.

### FNAB-TG Levels

FNAB-TG levels in the 39 cases diagnosed as metastatic PTC by cytology ranged from < 0.3–195200 ng/ml (median 312 ng/ml, normal < 10 ng/ml). In 28 cases (72%) TG levels were ≥ 10 ng/ml (mean 17267.75); histopathologic confirmation of metastatic PTC was available in 25 cases; no histologic follow-up was available in the remaining 3 cases. Of the eleven cases (28%) with TG levels < 10 ng/ml, 7 cases were diagnosed as PTC-TCV, 3 as classic-PTC and one as metastatic well-differentiated follicular derived carcinoma on histological follow-up. In this group one case of metastasis from PTC had a previous diagnosis of follicular carcinoma with a 0.7 cm focus of papillary microcarcinoma.

In 35 cases diagnosed as no tumor seen (NTS) on cytology (n = 35) FNAB-TG levels ranged from < 0.15–411900 ng/ml (median 0.3 ng/ml). In this group 30 cases (86%) had TG levels < 10 ng/ml. All 5 cases diagnosed as NTS with TG levels ≥ 10 ng/ml underwent surgery and on histological follow-up 3 were diagnosed as classic PTC, and 2 as FVPTC.

In the 23 cases diagnosed as non-diagnostic/inadequate due to lack of lymphoid or epithelial cells (NDX) on cytology FNAB-TG levels ranged from < 0.15–22600 ng/ml (median 0.3 ng/ml). Nineteen out of the 23 (83%) NDX cases had TG levels < 10 ng/ml. Of the remaining 4 cases (17%) that had TG levels ≥ 10 ng/ml, three were diagnosed as PTC on histology, and one had no follow-up.

In the group of 14 cases diagnosed as atypical/suspicious for PTC on cytology the FNAB-TG levels ranged from < 0.3–42560 ng/ml (median 14.9 ng/ml). Five of these cases (36%) had TG levels < 10 ng/ml and were diagnosed as PTC (n = 3) and PTC-TCV (n = 1) upon histological examination. There was no follow-up available in one case. Of the 9 cases (64%) with TG levels ≥ 10 ng/ml surgical follow-up showed PTC (n = 6), PTC-TCV (n = 1), and FVPTC (n = 2).

In the remaining three cases FNAB TG levels were: 2.8 ng/ml in the case with the cytological diagnosis of paraganglioma, 1272 ng/ml in case diagnosed as poorly differentiated carcinoma (PDCA) and 0.3 ng/ml in case diagnosed as carcinoma not otherwise specified (Table 1).

**Table 1:** Summary of FNAB diagnoses with histological follow-up and FNAB-TG levels correlation

	***SURGICAL PATHOLOGY FOLLOW-UP***					
	***TG Levels ≥ 10 ng/ml***			***TG Levels ≤ 10 ng/ml***		

***Cytologic-DX***						

	PTC	Other CA	No F/U	PTC	Other CA	No F/U

***PTC (n = 39)***	25	0	3	10*	1**	0
***NTS (n = 35)***	5	0	0	0	0	30
***NDX(n = 23)***	3	0	1	0	0	19
***ATYP (n = 15)***	9	0	0	4*	0	1
***OTHER (n = 3)***	0	1***	0	0	1****	0

DX = Diagnosis, TG = Thyroglobulin, F/U = surgical pathology follow- up, PTC = Papillary thyroid carcinoma, CA = Carcinoma, NTS = No tumor seen, NDX = Non-diagnostic, ATYP = Atypical/Suspicious, * = includes cases of tall cell variant of papillary carcinoma, ** = metastatic well-differentiated follicular derived carcinoma *** = poorly differentiated carcinoma, **** = carcinoma not otherwise specified.

## Discussion

The well-differentiated thyroid carcinoma (WDTC) after surgical excision and radioiodine ablation is usually followed by basal and TSH stimulated serum TG measurement, ^131^I whole body scan and neck ultrasound [20,21]. Since, well differentiated thyroid tumor cells retain their functionality, elevated serum TG levels are often indicative of residual tissue in thyroid bed or metastatic tumor [22-24]. Tumor recurrences in WDTC are often encountered in thyroid bed, lateral and central neck nodes [23-25]. The pathologic diagnosis of which is often required before submitting the patient to further surgical exploration because reactive/hyperplastic lymph nodes, reactive scar tissue and rarely post-surgical traumatic neuroma can mimic tumor recurrences on ultrasound examination [11,14,16].

Ultrasound guided fine-needle aspiration biopsy (US-FNAB) can effectively diagnose tumor recurrences in > 90% of cases on the basis of morphology alone, however, false negative results can be seen in 6–8% of cases. This could be due to absence of tumor cells in the FNAB specimen, partial or focal involvement of the lymph node or extensive tumor cell degeneration in some cases of PTC [11-19,26]. Measurements of TG in the rinse of the aspiration biopsy needle have been proposed for detection of neck lymph node metastasis from WDTC in patients after total thyroidectomy and radioiodine ablation [11-19,26].

Pacini et al in 1992 first reported the use of TG measurements in FNAB specimens (FNAB-TG) of non-thyroidal neck masses in 35 patients. In this study FNAB-TG levels diagnosed metastatic thyroid cancer in 14 patients, which was confirmed by histopathologic follow-up. These authors concluded that FNAB-TG had better negative predictive value than cytology alone [11]. Cignarelli et al reported FNA-TG to be more sensitive than FNAB alone especially in cases where cystic degeneration of metastatic deposits in neck nodes may contain few degenerated or no tumor cells [15]. Similar results have been documented by various studies from other insititutions [12-14,16-19,26].

In the present study we analyze the usefulness of TG levels as an adjuvant to FNAB in the diagnosis of lymph nodes suspicious for recurrence of PTC under ultrasound in 115 cases in 89 patients with history of previous thyroid carcinoma. When analyzing our results some discrepancies between the cytological diagnosis and the expected TG level were noted. Of the eleven cases (11/39 28%) diagnosed as PTC and 4 cases (4/15 27%) diagnosed as atypical/suspicious for PTC on cytology with TG levels < 10 ng/ml, eight cases (8/15 53%) had a diagnosis of PTC-TCV on histologic follow-up. The PTC-TCV is an aggressive variant of PTC. Its pathologic diagnosis is dependent upon the characteristic morphology i.e. presence of elongated cells with height being three times the width, oncocytic cytoplasm and nuclear features of PTC. It has been shown that these tumors can be difficult to treat because of their loss of iodine-avidity and decreased or absence of thyroglobulin production; this is especially encountered in recurrent tumors [26].

The limitations of FNAB, particularly in the setting of cystic lymph nodes where sampling of diagnostic areas is potentially problematic, were highlighted by the correlation with TG levels. All of the 5 cases diagnosed as no tumor seen on cytology with TG levels ≥ 10 ng/ml resulted in PTC on surgical follow-up. Similarly, 3 cases considered non-diagnostic on cytology due to lack of any cells with increased TG levels resulted in PTC diagnoses on follow-up.

Particularly important are the results in the category of atypical/suspicious for PTC recurrence. The cases with TG levels ≥ 10 ng/ml resulted in the diagnoses of PTC on follow-up; however, all cases with TG levels within normal limits also resulted in diagnosis of malignancy. This led us to believe that the cytopathology findings should rule over TG levels especially in cases of atypical/suspicious cytologic results. TG levels will not aid in the distinction between benign and malignant if a cytological specimen is atypical enough to warrant this diagnosis.

The diagnostic value of FNAB and FNAB-TG could not be calculated in this study due to lack of histologic follow-up in majority of cases diagnosed as no tumor seen and non-diagnostic.

The presence of serum anti-TG antibodies (TG-Ab) which can occur in up to 25–30% of patients can seriously affect serum TG measurements leading to either false positive or false negative results [27,28]. Therefore some authors have excluded patients with serum TG-Ab from their studies on the role of TG-FNAB in the diagnosis of recurrent WDTC [14]. Recently reports by Baskin et al and Boi et al have shown that TG-FNAB measurements are not affected significantly by the presence of TG-Ab in the FNAB specimen [16,18]. In view of these studies we did not include the presence or absence of TG-Ab in this study.

In summary, we have demonstrated similar to other studies that TG can be effectively measured in FNAB specimens from lymph nodes. FNAB-TG measurement is a useful technique for the diagnosis of lymph node metastasis originating from well-differentiated thyroid carcinoma, particularly when confronted with abnormal-appearing lymph nodes of small size or that have undergone cystic change.
